# Comprehensive bioinformatics analysis of the solute carrier family and preliminary exploration of SLC25A29 in lung adenocarcinoma

**DOI:** 10.1186/s12935-023-03082-7

**Published:** 2023-09-29

**Authors:** Pengdou Zheng, Zhenyu Mao, Miao Luo, Ling Zhou, Lingling Wang, Huiguo Liu, Wei Liu, Shuang Wei

**Affiliations:** 1 Department of Respiratory and Critical Care Medicine, Key Laboratory of Pulmonary Diseases of Health Ministry, Tongji Hospital, Tongji Medical College, Huazhong University of Science and Technology, 430030 Wuhan, China; 2 Department of Geriatrics, Tongji Hospital, Tongji Medical College, Huazhong University of Science and Technology, 430030 Wuhan, China

**Keywords:** Lung adenocarcinoma, SLC25A29, Prognostic model, Solute carrier family, Histone lactylation

## Abstract

According to the latest epidemiological investigation, lung adenocarcinoma (LUAD) is one of the most fatal cancer among both men and women. Despite continuous advancements in treatment approaches in recent years, the prognosis for LUAD remains relatively poor. Given the crucial role of the solute carrier (SLC) family in maintaining cellular energy metabolism stability, we conducted a comprehensive analysis of the association between SLC genes and LUAD prognosis. In the present study, we identified 71 genes among the SLC family members, of which 32 were downregulated and 39 were upregulated in LUAD samples. Based on these differentially expressed genes, a prognostic risk scoring model was established that was composed of five genes (*SLC16A7*, *SLC16A4*, *SLC16A3*, *SLC12A8*, and *SLC25A15*) and clinical characteristics; this model could effectively predict the survival and prognosis of patients in the cohort. Notably, *SLC2A1*, *SLC25A29*, and *SLC27A4* were identified as key genes associated with survival and tumor stage. Further analysis revealed that *SLC25A29* was underexpressed in LUAD tissue and regulated the phenotype of endothelial cells. Endothelial cell proliferation and migration increased and apoptosis decreased with a decrease in *SLC25A29* expression. Investigation of the upstream regulatory mechanisms of *SLC25A29* revealed that *SLC25A29* expression gradually decreased as the lactate concentration increased. This phenomenon suggested that the expression of *SLC25A29* may be related to lactylation modification. ChIP-qPCR experiments confirmed the critical regulatory role played by H3K14la and H3K18la modifications in the promoter region of *SLC25A29*. In conclusion, this study confirmed the role of SLC family genes in LUAD prognosis and revealed the role of *SLC25A29* in regulating endothelial cell phenotypes. These study results provided important clues to further understand LUAD pathogenesis and develop appropriate therapeutic strategies.

**Supplementary Information:**

The online version contains supplementary material available at 10.1186/s12935-023-03082-7.

## Highlights

Solute carrier family effectively predict the prognosis of LUAD patients.

Lactic acid decreases SLC25A29 transcription in endothelial cell.

Lactic acid in endothelial cells is mainly derived from the tumor microenvironment.

The decreased SLC25A29 expression is associated with H3K14la and H3K18la modifications.

Lowering SLC25A29 increases endothelial cell proliferation and migration.

**Supplementary Information:**

The online version contains supplementary material available at 10.1186/s12935-023-03082-7.

## Introduction

The most recent data from the American Cancer Society in 2023 reported that lung cancer accounts for approximately 238,340 new cases and 127,070 deaths, being the leading cancer in both men and women, excluding cancers of the reproductive system [1]. Notably, lung cancer has the highest mortality rate among both genders. LUAD is the most common subtype of non-small cell lung cancer and accounts for a significant proportion of lung cancer cases worldwide [2]. Despite significant advancements in LUAD treatment over the past few decades [3–6], the prognosis of patients remains grim, with a mere 4–17% 5-year survival rate [7]. Hence, investigating this situation at the molecular level to enable precise diagnosis, effective treatment, and accurate prognosis assessment is crucial.

The SLC gene family, also known as the solute carrier superfamily, comprises a diverse group of membrane transport proteins required for transporting a wide array of substances across cellular membranes. These proteins participate in ion, amino acid, sugar, nucleotide, vitamin, neurotransmitter, and drug transportation [8]. Moreover, the SLC gene family plays a significant role in cancer development and progression, as alterations in the expression and function of SLC transporters have been observed in various types of cancer [9, 10]. These changes affect tumor progression by influencing crucial processes, such as nutrient transport, drug resistance, metabolite transport, and pH regulation [11–13]. In particular, proton-coupled monocarboxylate transporters MCT1–4 facilitate the transmembrane movement of essential monocarboxylates in metabolism and have emerged as targets for cancer therapy due to their enhanced expression in various tumors [14]. SLC5A7 inhibits tumor growth by directly interacting with p53 and modifying p53 in colorectal cancer, thereby disrupting the interaction between p53 and MDM2 and promoting p53 protein expression [10]. In summary, SLC family genes should be included in the prognostic model. Currently, studies on prognostic models of SLC in LUAD have been conducted by identifying differentially expressed genes (DEGs) in nonpaired LUAD samples and establishing prognostic models. However, the predictive accuracy of these models is relatively low, ranging from 0.615 to 0.700, indicating the need for higher accuracy prognostic models [15].

Furthermore, *SLC25A29* was recognized for its exceptional performance in this study. *SLC25A29*, also known as the solute carrier family 25 member 29, is a gene that encodes a mitochondrial solute carrier protein. *SLC25A29* is expressed in various tissues, with higher levels observed in the heart, liver, and skeletal muscle. This protein belongs to the SLC25 family, which is involved in transporting various molecules across the inner mitochondrial membrane [16]. The primary physiological function of *SLC25A29* is the transport of arginine, lysine, homoarginine, methylarginine, and to a lesser extent, ornithine and histidine. These amino acids are used in mitochondrial protein synthesis and amino acid degradation. However, the relationship with pathological conditions and *SLC25A29* remains unclear [17, 18]. Given the importance of *SLC25A29* in mitochondrial function and its potential implications in various diseases, further research on its precise mechanisms of action is warranted.

## Results

### Overview of DEGs in SLC in LUAD

**Fig. 1:**
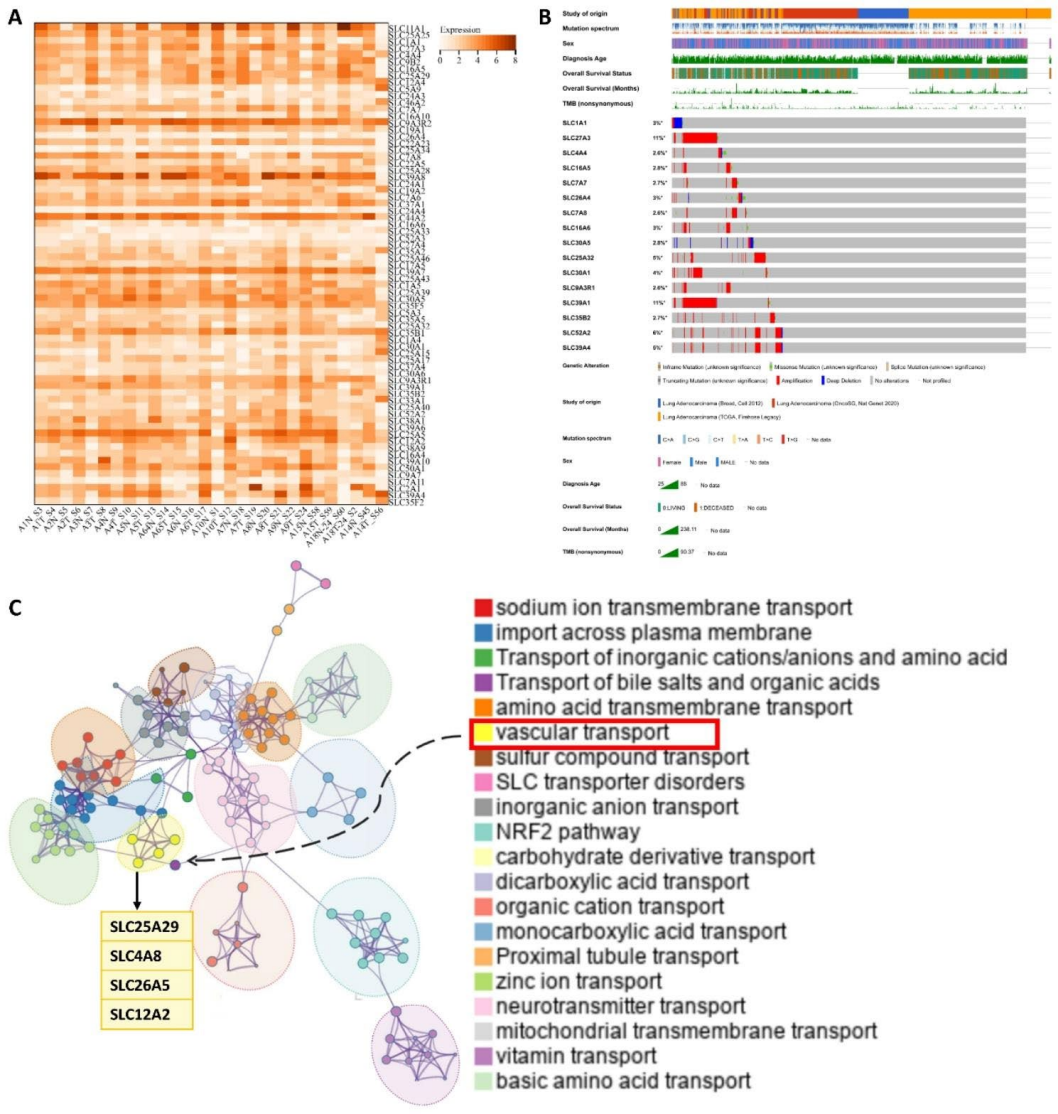
Expression of SLC in LUAD. (**A**) The heatmap showed the expression of 71 SLC members with significant differences between paired LUAD and adjacent normal tissues in the GSE159857 dataset. (**B**) The mutation landscape of the top 16 genes with the highest mutation rate among 71 DEGs was preliminarily demonstrated by cBioProtal. (**C**) Protein interaction network of SLC members. The colors of different nodes represented different functional groups, and the size of nodes indicated their importance in the network

We detected transcript sequencing data of 369 SLC family members from the GSE159857 dataset, including those of 12 LUAD samples and associated adjacent normal lung tissue samples. Limma analysis of paired samples (Fig. 1A) was used to identify 71 DEGs within the SLC family in LUAD. Of these, 32 genes exhibited significant downregulation, whereas 39 genes displayed significant upregulation. We analyzed somatic mutations and DNA copy number changes using cBioPortal to investigate the genetic alterations in these DEGs. The analysis revealed that *SLC39A1* and *SLC27A3* were the most frequently mutated genes, with mutations occurring at a rate of 11% in each (Fig. 1B).

Furthermore, we used Metascape to explore the protein–protein interactions of these DEGs. The analysis revealed the formation of distinct modules based on their functional characteristics, with notable interconnections between different modules. Primarily, these proteins are involved in transporting amino acids, nucleotides, sugars, and inorganic ions, among other substances. Notably, certain genes (*SLC25A29*, *SLC4A8*, *SLC26A5*, and *SLC12A2*) within specific functional modules were found to be associated with blood vessels (Fig. 1C).

### Establishment of a nomogram to predict overall survival (OS) based on patient information in the Cancer Genome Atlas (TCGA) cohort

**Fig. 2:**
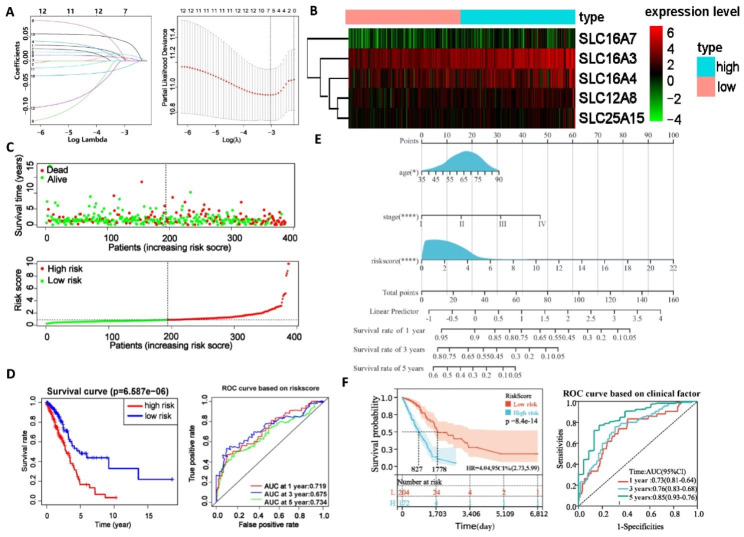
Construction of a prognostic risk scoring model. (**A**) Coefficient distribution and error distribution of LASSO regression. (**B**) Heatmaps of the expression of five genes included in the risk score model. (**C**) Survival time and status profiles of patients with varying risk scores. (**D**) and (**F**) Kaplan-Meier survival analysis was performed to calculate the OS of high-risk and low-risk groups patients in the TCGA dataset. The ROC curve was used to verify the ability of the risk model to judge the 1-, 3-, and 5-year OS rates of patients in the TCGA. (**E**) The nomogram, including risk scores and clinical features, showed the prognosis estimates of the cases in the TCGA database. ns, nonsignificant, *P < 0.05, **P < 0.01, ***P < 0.001, ****P < 0.0001

To develop a new risk score model consisting of prognostic DEGs, we performed univariate Cox regression, lasso regression, and multivariate Cox regression analyses. Through these analyses, we identified 13, 7, and 5 DEGs, respectively, based on the 95% confidence interval of the genes (Supplementary Tables 1 and 2, Fig. 2A). The five selected DEGs were *SLC16A7*, *SLC16A4*, *SLC16A3*, *SLC12A8*, and *SLC25A15*. These five prognostic-related genes were used to establish a prognostic risk scoring model with the following formula: Risk score = Exp_SLC16A7_ × 0.092 + Exp_SLC16A4_ × 0.014 + Exp_SLC16A3_ × 0.021 + Exp_SLC12A8_ × 0.037 + Exp_SLC25A15_ × 0.065. Subsequently, the aforementioned formula was used to calculate the risk scores for each patient in the TCGA cohort. Patients were then categorized into low-risk and high-risk groups based on the median risk score (Fig. 2B). The expression of these five DEGs in the high-risk and low-risk groups was visualized through a heatmap; the expression levels of these DEGs in the high-risk group were higher than those in the low-risk group. Furthermore, a scatter plot demonstrated a significant decrease in survival time of patients with higher risk scores, accompanied by an increased number of patients in the “dead” state (Fig. 2C). Kaplan–Meier survival analysis confirmed that patients at high risk, who are classified by their risk scores, exhibited shorter OS and a poorer prognosis than patients at low risk. The area under the receiver operating characteristic curve (ROC) for 1-year, 3-year, and 5-year OS was 0.719, 0.675, and 0.734, respectively, indicating the model’s effectiveness in predicting OS in TCGA cases (Fig. 2D).

To further improve the prediction efficacy of the model, three clinical features, namely, age, gender, and stage, were combined with the risk score model for independent prognostic factor analysis (Supplementary Tables 3 and 4). Eventually, age, stage, and risk scores were incorporated into the nomogram (Fig. 2E). Kaplan–Meier survival analysis revealed that patients at high risk experienced a relatively short OS duration (hazard ratio [HR] = 4.04, *P* < 0.01). ROC analysis revealed that the nomogram was more effective than the single risk score model in predicting OS at 1, 3, and 5 years, with area under the curve (AUC) values of 0.73, 0.76, and 0.85, respectively (Fig. 2F). These results suggested that the SLC-based nomogram is a reliable prognostic predictor for patients with LUAD.

### Association between expression of DEGs and clinical features of patients

**Fig. 3:**
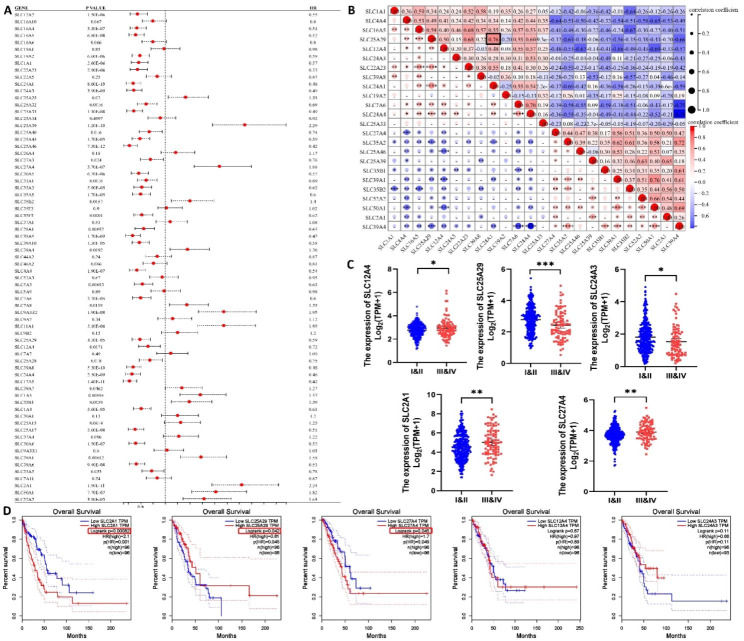
Association between expression of SLC members and clinical features of patients. (**A**) Forest map of associations between expression of 71 DEGs and OS in LUAD patients. (**B**) Spearman’s rank correlation analyses between 24 genes associated with survival at the transcriptional level. Negative and positive correlations were marked blue and red, respectively. Both the size and color of the dots indicated the strength of the correlation. The black box showed the two genes with the strongest positive correlation. Correlation analysis of SLC12A4, SLC25A29, SLC24A3, SLC2A1, SLC27A4 with clinical stage (**C**) and OS (**D**) in TCGA database. ns, nonsignificant, *P < 0.05, **P < 0.01, ***P < 0.001, ****P < 0.0001

To understand the relationship between DEGs and clinical characteristics of patients, we focused on OS and clinical stage. Kaplan–Meier analysis of 1308 LUAD cases from GEO and EGA revealed that 50 of 71 DEGs were associated with OS (Fig. 3A). Upregulated genes that were detrimental to OS included *SLC39A4* (HR = 1.36), *SLC35B2* (HR = 1.4), *SLC35B1* (HR = 1.39), *SLC39A1* (HR = 1.58), *SLC52A2* (HR = 1.63), *SLC50A1* (HR = 1.82), *SLC27A4* (HR = 1.88), *SLC2A1* (HR = 2.24), and *SLC25A39* (HR = 2.29), whereas downregulated genes that were beneficial to OS included *SLC25A46* (HR = 0.42), *SLC24A4* (HR = 0.46), *SLC24A1* (HR = 0.48), *SLC24A3* (HR = 0.49), *SLC25A33* (HR = 0.49), *SLC16A5* (HR = 0.52), *SLC4A4* (HR = 0.54), *SLC22A23* (HR = 0.55), *SLC1A1* (HR = 0.57), *SLC25A29* (HR = 0.59), *SLC19A2* (HR = 0.59), *SLC7A6* (HR = 0.6), *SLC35A2* (HR = 0.62), and *SLC12A4* (HR = 0.72). Supplementary Table 5 provides a brief list of metabolic substrates transported by the abovementioned 24 DEGs. The positive (red) and negative (blue) correlations among these 24 DEGs at the transcription level were examined using Spearman rank correlation test (Fig. 3B). *SLC24A1* and *SLC25A29* had the strongest positive correlation, with a correlation coefficient of 0.76, whereas *SLC39A4* and *SLC24A4* had the strongest negative correlation, with a correlation coefficient of − 0.78. Correlation analysis indicated that *SLC12A4*, *SLC25A29*, *SLC24A3*, *SLC2A1*, and *SLC27A4* were associated with disease staging (Fig. 3C), whereas the remaining 19 DEGs showed no significant correlation (Supplementary Fig. 1A). Further survival analysis based on TCGA data confirmed that *SLC2A1*, *SLC25A29*, and *SLC27A4* were associated with OS (Fig. 3D). Supplementary Fig. 1B shows the immunohistochemical staining images for *SLC2A1*, *SLC25A29*, and *SLC27A4* of LUAD and paracancer normal tissues obtained from the HPA database, and the results were as expected.

### *SLC25A29* was mainly expressed in endothelial cells and is associated with angiogenesis

**Fig. 4:**
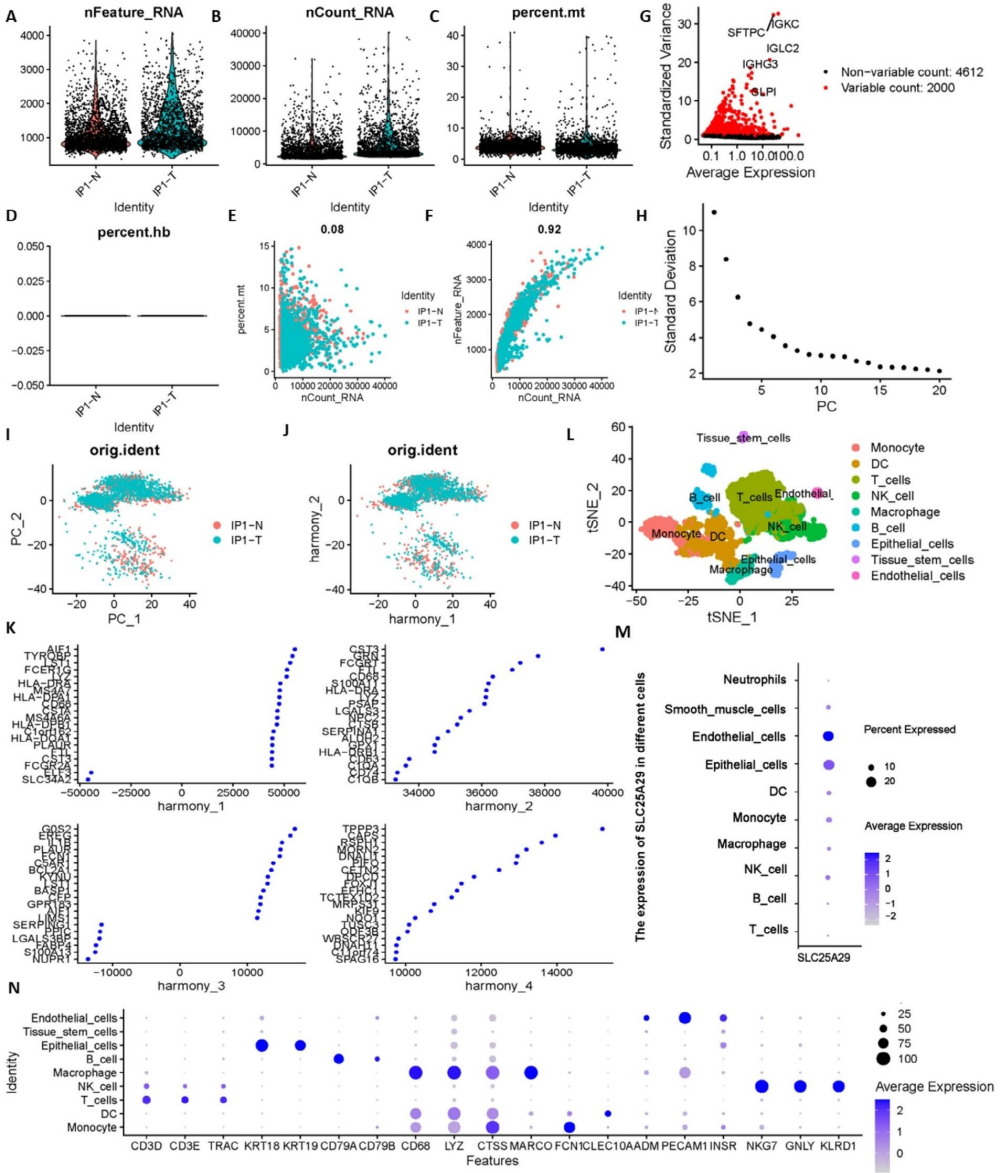
Single-cell RNA sequencing analysis identified that SLC25A29 was primarily expressed in endothelial cells. The number of genes that could be detected in all cells (**A**), the transcript of each gene (**B**), the percentage of mitochondrial genes (**C**), and the percentage of erythrocyte genes (**D**) in the samples before quality control. After quality control and data filtering, correlation analysis was conducted between sequencing depth and percentage of mitochondrial genes (**E**), and between sequencing depth and detected genes (**F**), and Pearson correlation coefficients were 0.08 and 0.92, respectively. (**G**) Volcano map of genes that were differentially expressed between cells. 2000 genes marked with the red dots had high variation and 4612 genes marked with the black dots had low variation. (**H**) The scree plot identified the top 10 PCs recommended for inclusion in subsequent analysis. (**I**) and (**J**) The harmony algorithm removes batch effects between samples. (**K**) The dot plot showed the top 20 significantly related genes in each harmony. (**L**) Combining the results of singleR auto-annotation and manual annotation based on marker genes (**N**), all cells were classified into nine broad categories. (**M**) SLC25A29 was mainly expressed in endothelial

Previously, we found vascular-related functional groups, namely *SLC25A29*, *SLC4A8*, *SLC26A5*, and *SLC12A2*. Subsequently, we performed a correlation analysis of DEGs and clinical features and screened out *SLC2A1*, *SLC25A29*, and *SLC27A4* that were associated with both OS and stage. The intersection gene *SLC25A29* attracted our attention and prompted us to perform a comprehensive analysis. First, the results of scRNA-seq were analyzed to further investigate the distribution of *SLC25A29* in LUAD. A total of 2,495 and 1,832 genes were initially detected in LUAD and paracancer tissues, respectively. After quality control and standardization of the scRNA-seq data, 2,481 and 1,792 high-quality cells from the sample core were included in the follow-up analysis of this study (Fig. 4A, B, C, and D). Correlation analysis revealed that sequencing depth was not related to the percentage of mitochondrial genes but was significantly positively related to the amount of RNA detected (Fig. 4E and F). To differentiate among cell types in tissues, we identified 2000 genes that were highly variable among cells via analysis of variance (Fig. 4G). In addition, principal component (PC) analysis was used to reduce the dimension of the data, and the first 10 PCs were selected following the suggestion of a scree plot (Fig. 4H). The scatter plot revealed a significant batch effect between samples, and the harmony algorithm was used to reduce this error (Fig. 4I and J). The top 20 related genes from PCs are shown as dot plots in Fig. 4K. The TSNE algorithm visualized the annotated cell population (Fig. 4L). The main types of cells visualized were monocytes, dendritic cells, T cells, NK cells, macrophages, B cells, epithelial cells, tissue stem cells, and endothelial cells, and their marker genes are shown in Fig. 4N. The bubble diagram revealed that *SLC25A29* was mainly expressed in endothelial cells, followed by epithelial cells and macrophages (Fig. 4M). The median *SLC25A29* expression value was used as the boundary to divide the TCGA cases into SLC25A29 high expression and SLC25A29 low expression group. Difference analysis revealed 5,314 statistically significant genes (SDEGs). These genes were mainly involved in molecular functions, such as carbohydrate binding and glycosaminoglycan binding, as well as cell components, such as cilia, microtubules, and extracellular matrix (Supplementary Fig. 2A and 2B). Functional analysis revealed that the *SLC25A29* low-expression group was closely associated with angiogenesis (Supplementary Fig. 2 C and D).

### Validation of functional phenotypes of *SLC25A29* in HUVEC

**Fig. 5:**
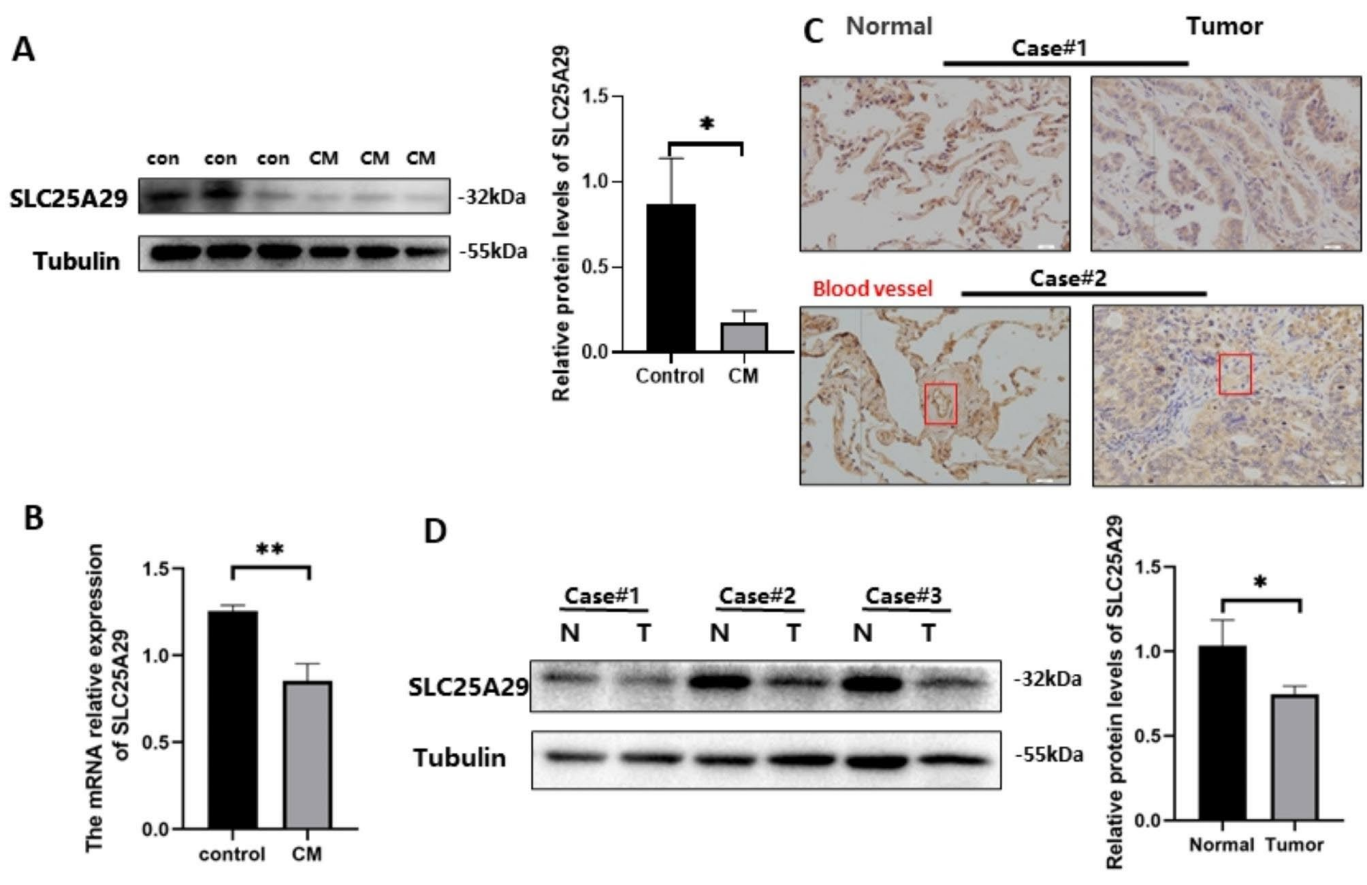
The expression of SLC25A29 was downregulated in LUAD. (**A**) and (**B**) The protein and mRNA expression levels of SLC25A29 in HUVEC. (**C**) Immunohistochemical staining of SLC25A29 in LUAD. (**D**) The protein levels of SLC25A29 in LUAD

**Fig. 6:**
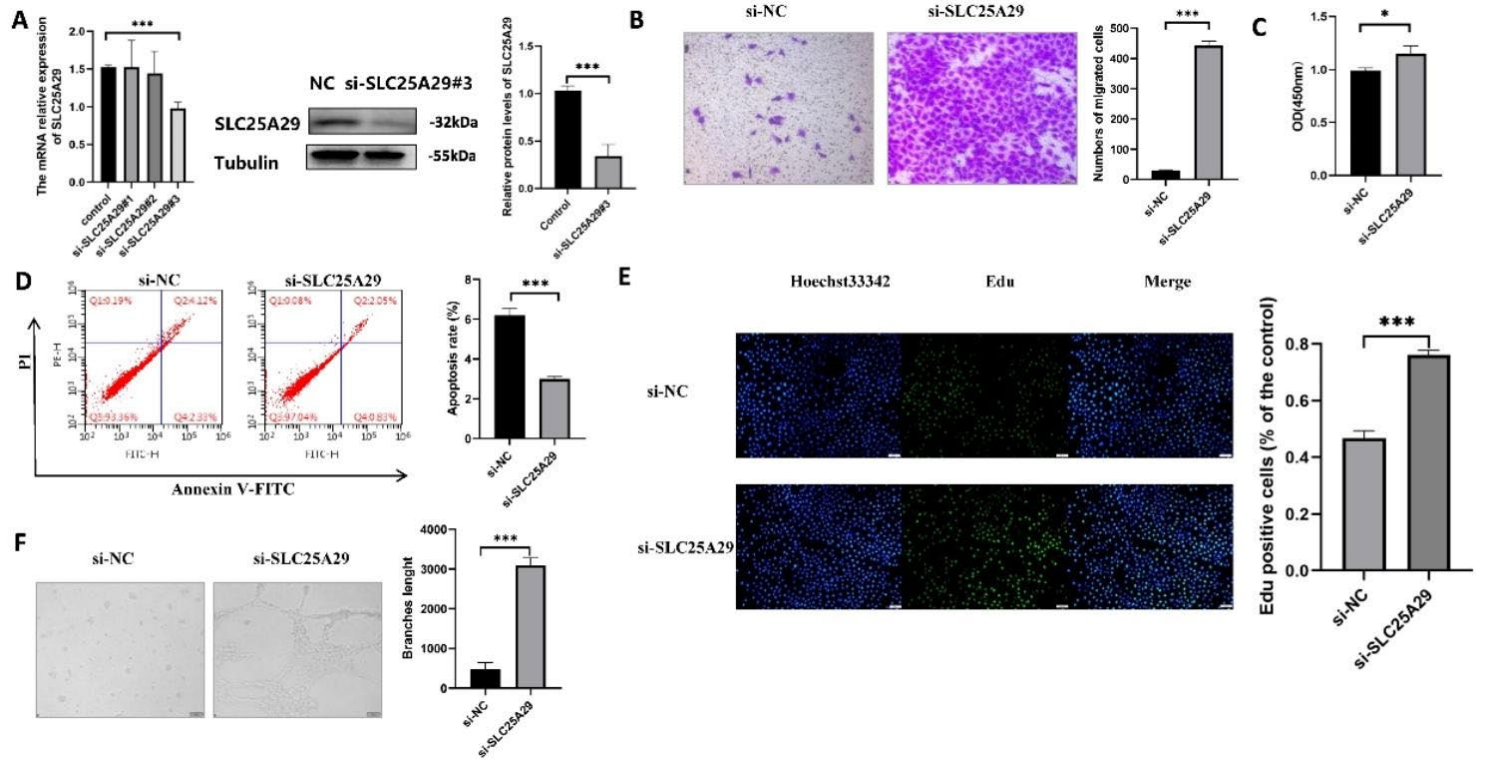
Effect of SLC25A29 knockdown on endothelial cell phenotype. (**A**) The knockdown effect of siRNA was verified by rt-PCR and western blot. (**B**) Transwell investigated the effect of SLC25A29 on cell migration. (**C**) CCK8 and (**E**) EDU assays evaluated the effects of SLC25A29 on cell proliferation. (**D**) The effect of SLC25A29 on apoptosis was verified by flow cytometry. (**F**) The angiogenesis capacity of SLC25A29 on HUVEC was evaluated in vitro

Preliminary analysis indicated that *SLC25A29* was underexpressed and was mainly expressed in endothelial cells. *SLC25A29* expression levels in HUVEC were detected via RT-qPCR or western blotting and compared between the CM and control groups (Fig. 5A and B). Immunohistochemistry revealed that *SLC25A29* was expressed at a lower level in endothelial cells in LUAD tissues than in adjacent normal tissues (Fig. 5C). The downregulation of *SLC25A29* in tumor tissue was demonstrated in three pairs of patients with LUAD protein samples (Fig. 5D). To verify the functional phenotype of *SLC25A29* in HUVEC, we designed three siRNA duplexes and found that si-SLC25A29#3 could effectively interfere with *SLC25A29* expression at the RNA level via RT-qPCR. Western blotting further confirmed that #3 effectively reduced the expression of *SLC25A29* at the protein level (Fig. 6A). Transwell experiments revealed that downregulated *SLC25A29* significantly promoted HUVEC migration (Fig. 6B). In addition, the decreased expression of *SLC25A29* significantly increased the proliferative activity of the cells and reduced their apoptosis (Fig. 6 C, D, and E). Furthermore, in vitro angiogenesis experiments confirmed that *SLC25A29* knockdown promoted angiogenesis (Fig. 6F). Overall, these results indicate the regulatory role of *SLC25A29* in angiogenesis.

### Regulation of *SLC25A29* expression via lactate modification

**Fig. 7:**
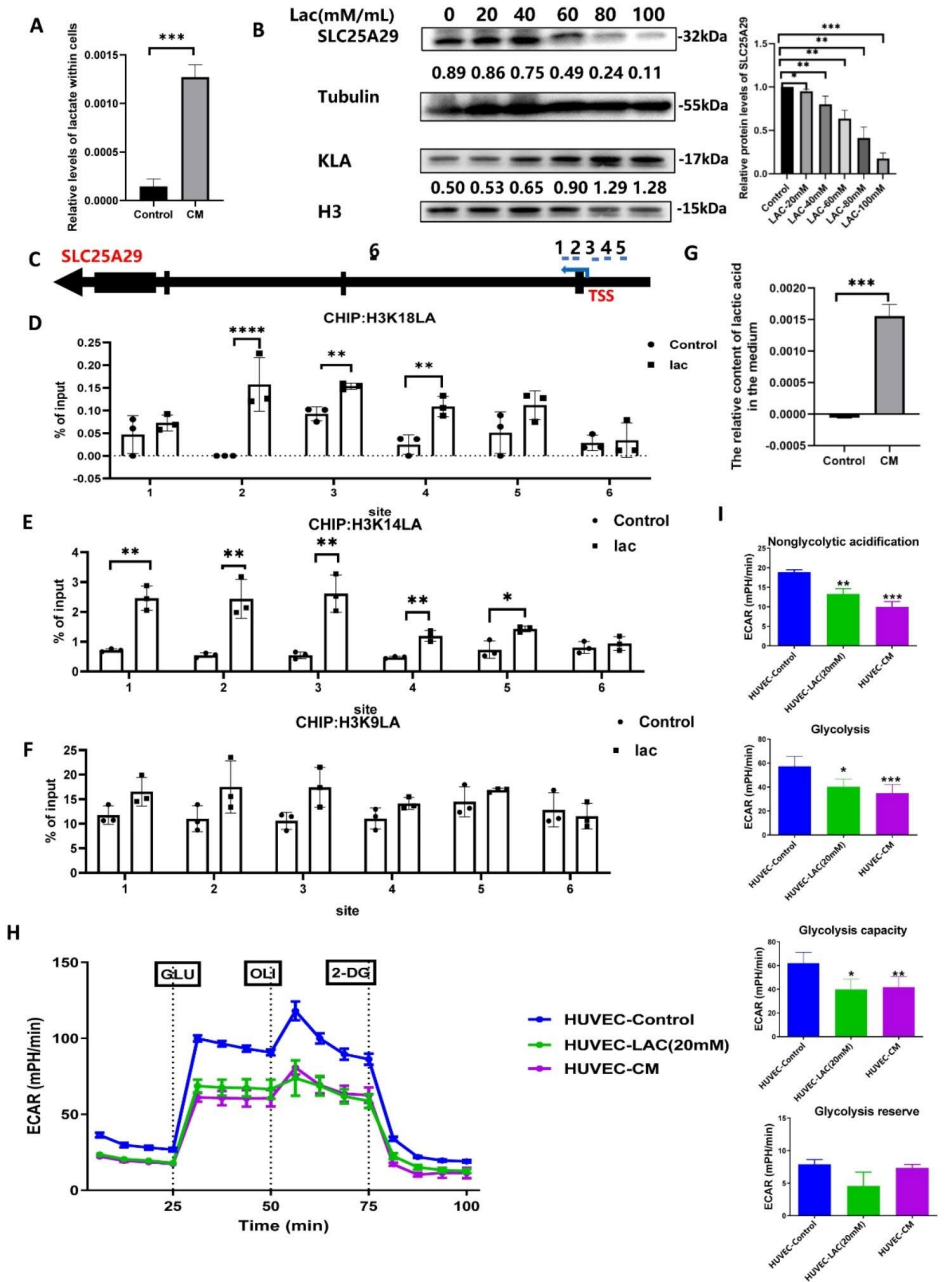
The down-regulation of SLC25A29 expression was related to lactylation modification in HUVEC. (**A**) Lactate content in HUVEC. (**B**) Western blot was performed to test the relationship between SLC25A29 expression levels and lactate. (**C**) Distribution of six primers on the SLC25A29 genomic sequence. (**D**) ChIP-qPCR assay of H3K18la status in the SLC25A29 genomic region in HUVEC. (**E**) ChIP-qPCR assay of H3K14la status in the SLC25A29 genomic region in HUVEC. (**F**) ChIP-qPCR assay of H3K9la status in the SLC25A29 genomic region in HUVEC. (**G**)Relative lactate content within the culture medium. (**H**) and (**I**) ECAR of HUVEC under different cultivation conditions

**Fig. 8:**
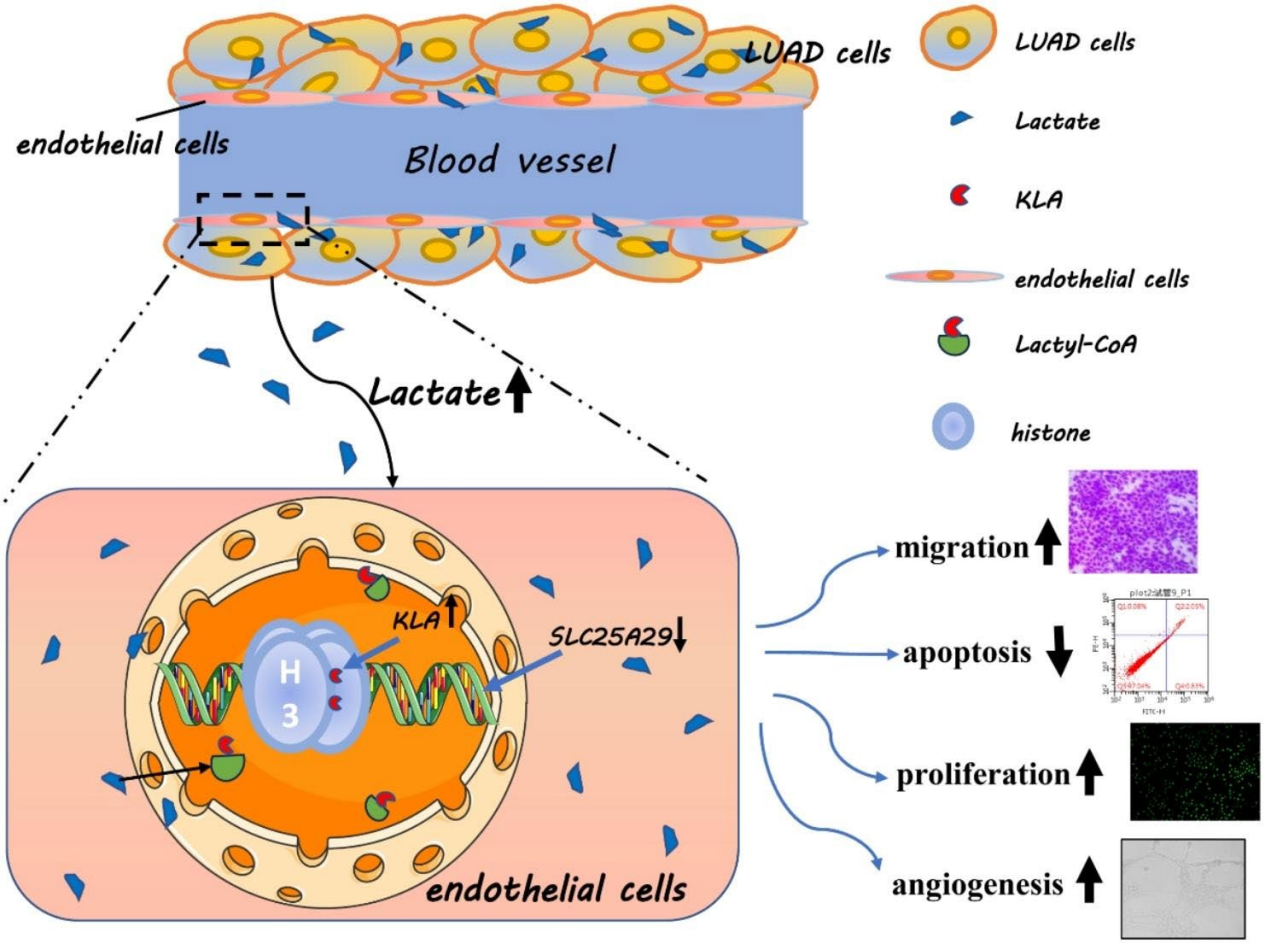
Regulatory pattern diagram of SLC25A29 in LUAD

To understand the reasons for the decreased expression of *SLC25A29* in the tumor microenvironment, we attempted to find some clues from 515 RNA-seq data in the TCGA database. We identified 156 genes with correlation coefficients above 0.4 and 841 genes with correlation coefficients below − 0.4 via correlation analysis (Supplementary Fig. 3A and B). Enrichment analysis of the genes negatively correlated with *SLC25A29* revealed that energy metabolism pathways and nonspecific pathways, such as aminoacyl-tRNA biosynthesis, were enriched (Supplementary Fig. 3C). Therefore, we measured the lactate content in the cell. The data revealed significantly increased lactate content in HUVEC cultured in CM (Fig. 7A). The expression of histone lysine lactate acylation (KLA) increased with increasing lactate concentration, whereas the expression of *SLC25A29* decreased gradually (Fig. 7B). We hypothesized that KLA modified histones in the *SLC25A29* region, thereby affecting *SLC25A29* expression. The results of chip-seq in the GEO database confirmed the presence of KLA modification in the promoter region of *SLC25A29* (Supplementary Fig. 4A and B). Six primers were designed based on the distribution of peaks in the promoter region (Fig. 7C). ChIP-qPCR experiments demonstrated that the promoter region of *SLC25A29* exhibited H3K18la and K3K14la enrichment, and this enrichment was observed to increase upon the addition of lactate. However, no significant changes were observed in H3K9la (Fig. 7D, E, and F). To identify the source of lactate, its concentration in the medium was measured. The results indicated that the lactate content in CM was higher than that in ordinary medium (Fig. 7G). The extracellular acidification rate (ECAR) was directly measured using the Seahorse XF extracellular flux analyzer. As shown in Fig. 7H, cells cultured with exogenous low-concentration lactate or CM demonstrated significantly reduced ECAR compared with the control group. In particular, the overall acid production capacity, including nonglycolic acidification, glycolysis, and glycolysis capacity, was significantly decreased in cells cultured with exogenous low-concentration lactate or CM. However, no significant difference was detected between the exogenous low-concentration lactate group and CM culture group (Fig. 7I). Therefore, our study concluded that tumor cells in LUAD produced and secreted large amounts of lactate and were taken up by endothelial cells, which provided adequate nutrients to endothelial cells while dramatically changing histone lactylation levels. The elevated histone lactylation level in the promoter region of *SLC25A29* reduced the transcription of *SLC25A29*, which in turn affected the proliferation, migration, and apoptosis of endothelial cells (Fig. 8).

## Materials and methods

### Data processing of single cell RNA seq (scRNA-seq)

The scRNA-seq data of human primary LUAD and paracancer normal samples with a reading depth of 10× genomics based on Illumina NextSeq 500, accession numbers GSM3304007 and GSM3304008, were downloaded from the Gene Expression Omnibus (GEO, http://www.ncbi.nlm.nih.gov/geo/) database. Low-quality genes were excluded based on the criteria of fewer than ten cell expressions, fewer than 200 unique gene counts, or mitochondrial gene percentage exceeding 10%. Principal Component Analysis (PCA) [19] and t-Distributed Stochastic Neighbor Embedding (t-SNE) [20] were employed for data dimensionality reduction. The singleR algorithm was utilized for cell annotation of cell populations.

### Acquisition of bulk RNA-seq

RNA sequencing data of 594 LUAD patients and clinical data of 493 LUAD patients were downloaded from the TCGA data portal (https://portal.gdc.cancer.gov/) for the establishment of a prognosis model and correlation analysis. Additionally, transcriptome sequencing data of 12 pairs of LUAD tumors and adjacent tissues were obtained from the GSE159857 dataset in the GEO database for acquiring DEGs.

### Identification and enrichment analysis of DEGs

The expression of SLC genes in LUAD and normal lung tissues at the transcriptional level was investigated using the paired-sample limma package in R. Genes with log2 |FC| more than 1 and p-value less than 0.05 were identified as DEGs (this condition applies throughout the study). The Metascape (https://metascape.org/gp/index.html) database was utilized to examine the protein-level interactions of the DEGs. geneontology [GO, including cellular component (CC), biological process (BP), molecular function (MF)] and kyoto encyclopedia of genes and genomes (KEGG) enrichment analysis of the DEGs was performed using the WEB-based GEne SeT AnaLysis Toolkit (WebGestalt, http://www.webgestalt.org/).

### Cell culture and cell transfection

Human umbilical vein endothelial cells (HUVEC) were kindly provided by Tongji Hospital Department of General Surgery, while the PC9 cell line was purchased from Zhongqiao Xinzhou Co., Ltd (Shanghai, China). Both cell lines were cultured in Dulbecco’s Modified Eagle Medium (DMEM) (Gibco Laboratories, Grand Island, NY USA) supplemented with 10% fetal bovine serum (FBS) and incubated in a sterile culture chamber at 37 degrees Celsius with 5% CO2 concentration. Human-specific SLC25A29 siRNA (sense: GGCUCUCUACCUAUCCUGU) and negative control sequences were synthesized by General Biotics, Inc. (Anhui, China) to silence SLC25A29 expression in HUVEC. Lipofectamine 3000 (Invitrogen, Carlsbad, CA, USA) was used as the carrier to transfect siRNA with a final concentration of 50 nm/ml into HUVEC. After 48 h, the treated cells were collected for additional analysis.

### RNA extraction and RT-qPCR

Total RNA was extracted from HUVEC using Trizol reagent according to the reagent manufacturer’s instructions. A total of 1000ng RNA was reverse-transcribed using the cDNA synthesis kit. Subsequently, using cDNA as a template, qRT-PCR was run to assess mRNA expression of the target gene. The relative mRNA expression value was evaluated by 2^−ΔΔCT^ method. The primer sequences were synthesized by Sangon Biotech (Shanghai, China), and their specific sequence were as follows: ACTIN (human) forward 5’-GCAAGCAGGACTATGACGAG-3’, reverse 5’-CAAATAAAGCCATGCCAATC-3’; SLC25A29 (human) forward 5’-AGAGCGTGGAGAAGCCTCGTA-3’, reverse 5’-CGTTGATGAAGGTGAGCCCCAT-3’.

### Western blotting analysis

Total proteins in cells were extracted using RIPA buffer (Aspen Biological, Wuhan, China) and protease inhibitors (MedChemExpress, SNJ, USA). Protein concentration was measured using the BCA kit (Aspen). Equal amounts of protein were separated by 10% SDS-PAGE, transferred to polyvinylidene fluoride (PVDF) membranes (Millipore, Billerica, MA, USA), and blocked with milk at room temperature for 1 h. PVDF membranes were washed three times with TBST for 10 min each, and then incubated overnight at 4 °C with the appropriate primary antibody. Following another round of washing, the membranes were incubated with secondary antibodies at room temperature for 1 h. Finally, the membranes were developed using ChemiDoc XRS gel imaging system (Bio-Rad, Hercules, CA, USA). The antibodies used in this experiment are listed below: Anti-beta Tubulin (PTM-6414, PTMBIO, Zhejiang, China); Anti-L-Lactyl-Histone H3 (Lys18) (PTM-1427RM, PTMBIO, Zhejiang, China); Anti-L-Lactyl-Histone H3 (Lys14) (PTM-1414RM, PTMBIO, Zhejiang, China); Anti-L-Lactyl-Histone H3 (Lys9) (PTM-1419RM, PTMBIO, Zhejiang, China); Anti-L-Lactyl Lysine (PTM-1425, PTMBIO, Zhejiang, China); Anti-Histone H3 (PTM-6621, PTMBIO, Zhejiang, China); SLC25A29 (26663-1-AP, Proteintech, Wuhan, China).

### Immunohistochemical (IHC) analysis

The paraffin-embedded LUAD tissue sections were deparaffinized by placing the slides in xylene, followed by rehydration through a series of graded alcohols. An antigen retrieval step was typically performed to unmask the target antigen epitopes. This was achieved by subjecting the tissue sections to heat-induced epitope retrieval (HIER) using Tris-EDTA buffer under specific temperature and time conditions. To minimize non-specific binding of antibodies, the tissue sections were treated with BSA. Subsequently, the tissue sections were incubated with a primary antibody specific to SLC25A29. After incubation with the primary antibody, the tissue sections were washed with phosphate-buffered saline (PBS) to remove unbound primary antibody and any remaining contaminants. Finally, the tissue sections were incubated with the secondary antibody. These steps enabled the visualization and assessment of SLC25A29 expression in paraffin-embedded LUAD tissue sections.

### Cell counting kit-8 (CCK-8) assay

A total of 3000 cells were seeded in each well of a 96-well plate, with separate wells for SLC25A29 knockout cells and negative control cells. After 24 h of culture, the cells were incubated with CCK-8 solution for 1 h. Following the incubation period, the absorbance at a wavelength of 450 nm was measured using a spectrophotometer. This measurement provides an indication of cell viability or metabolic activity based on the colorimetric reaction of the CCK-8 reagent (Biosharp, BS350A, Anhui, China). The experiment was performed in triplicate, and representative results are presented.

### Gene mutation analysis

Utilize the cBioPortal platform (http://www.cbioportal.org/) to analyze the spectrum of SLC mutations, encompassing DNA mutations as well as alterations in mRNA expression levels.

### 5-Ethynyl-2′-deoxyuridine (EdU) assays

The BeyoClick™ EdU Cell Proliferation Kit with Alexa Fluor 488 (Beyotime, C0071S, Shanghai, China) was used following the manufacturer’s instructions. The treated cells were evenly distributed into 96-well plates, and EDU staining was conducted when the cells reached confluence. Nuclei were counterstained with DAPI. After treatment with the kit, proliferating cells exhibited intense green fluorescence when observed under a fluorescence microscope. Images were captured and analyzed using ImageJ software (version 1.5.3). The experiment was performed in triplicate, and representative results are presented.

### Apoptosis assessment

The Annexin V/PI Apoptosis Detection Kit (Key Gen, Nanjing, China) was used for quantitative analysis of cell death in HUVEC cells through flow cytometry. Cells were harvested with trypsin, washed with PBS, and suspended in 500 µl of binding buffer containing 5 µl of Annexin V-FITC and 5 µl of PI. After a 15-minute incubation in the dark at room temperature, flow cytometry analysis was performed using the Becton Dickinson LSR flow cytometer to detect the samples.

### Tubule formation experiment

Coat the cell culture plate with Matrigel (ABW, 082704, Shanghai, China) and place it in a 37-degree Celsius incubator for one hour. Subsequently, seed 30,000 HUVEC cells in each well and incubate for 8 h. Observe tubule formation using an inverted microscope. Analyze the images using ImageJ software to quantify the parameters of tubule formation.

### Extracellular acidification rate (ECAR)

The Seahorse XF Analyzer (Agilent technologies, XF24, California, USA) was used to measure ECAR. ECAR was a metabolic parameter that reflects the rate of extracellular acid production, mainly resulting from the glycolytic breakdown of glucose. The Seahorse XF Analyzer measured ECAR by monitoring the changes in the pH of the extracellular media over time. This measurement provided valuable insights into cellular glycolytic activity and lactate production, which were important indicators of cellular metabolism and energy production. The reagents used were as follows: XF Base Medium (Agilent technologies, 102,353); XF96 Cell Culture Microplates (Agilent technologies, 102,416); XF96 Extracellular Flux Assay Kits (Agilent technologies, Q29216); XF Calibrant (Agilent technologies, 100,840); XF Cell Mito Stress Test Kit (Agilent technologies, 103015-100).

### Chromatin immunoprecipitation followed by quantitative PCR (ChIP-qPCR)

The CHIP-qPCR (Cell Signaling Technology, 9003, Massachusetts, USA) procedure was carried out according to the instructions provided by the reagent manufacturer. In summary, HUVEC cells were fixed with 1% formaldehyde to cross-link the chromatin. The chromatin was then fragmented by sonication. Three CHIP-grade antibodies, H3K9la, H3K14la, and H3K18la, were used to enrich the protein-chromatin fragments. After washing to remove non-specific binding and contaminants, the chromatin fragments were eluted and purified. The purified DNA fragments were then subjected to quantitative PCR (qPCR) targeting specific genomic regions using primers designed based on the chip-seq data from the GEO databases GSE156675 and GSE212087. This approach allowed for the quantification of the enrichment of the target gene regions in the purified DNA fragments.

### Lactate measurement

The lactate levels in cells were detected using the CheKine™ Lactic Acid Content Test Kit (Abbkine, KTB1100, California, USA) following the manufacturer’s instructions. Lactate was extracted from cells by adding 200 µl of lactic acid buffer solution to each well of a 6-well plate. The extracted lactate was then mixed with 50 µl of working solution in equal proportion and incubated in a light-protected incubator at 37 °C for 30 min. Subsequently, the absorbance at 450 nm was measured using a multifunctional enzyme marker, and the lactic acid content was calculated based on the standard curve.

### HPA database

The protein expression of SLC25A29, SLC2A1, and SLC27A4 was assessed in both cancerous and normal tissues using the online tools provided by the Human Protein Atlas (HPA) at http://www.proteinatlas.org.

### Survival analysis

The survival data for LUAD patients were retrieved from the TCGA data portal. Overall survival (OS) was examined to explore the association between the expression of SLC members and patient prognosis. Prognostic evaluation of SLC members in cancer was performed using the “forestplot” and “survival” packages in R.

### Statistical analysis and data visualization

The results are presented as the mean ± standard deviation (SD). Inter-group analysis was conducted using GraphPad Prism 7.0, employing the t-test, and statistical significance was set at P < 0.05. Data were analyzed and visualized using R software [21] (Version 4.2.1; https://www.r-project.org/).

## Discussion

The present study aimed to investigate the DEGs in the SLC family in LUAD and their association with clinical features and patient prognosis. In total, 71 DEGs were identified among the SLC family members, of which 32 were downregulated and 39 were upregulated in LUAD samples. A prognostic risk scoring model based on the DEGs was established after further analysis and research.

This model consisted of five genes: *SLC16A7*, *SLC16A4*, *SLC16A3*, *SLC12A8*, and *SLC25A15*. SLC16A7, also known as monocarboxylate transporter 2 (MCT2), is a protein that belongs to the SLC16A family of transporters. It transports monocarboxylates, such as lactate, pyruvate, and ketone bodies, across cell membranes. MCT2 facilitates the transport of monocarboxylates across cell membranes by acting as a proton-linked transporter. It cotransports a monocarboxylate molecule along with a proton, utilizing the proton gradient across the membrane [22]. This process is essential for efficient energy metabolism and metabolic cooperation between different cell types. Studies have revealed that the dysregulation of MCT2 expression and function is associated with various diseases and conditions. In particular, altered MCT2 expression has been observed in certain cancers, neurodegenerative disorders, and metabolic disorders [23–25].

SLC16A4, also known as monocarboxylate transporter 5, is a protein that belongs to the SLC16A family of transporters. SLC16A4 primarily functions to transport monocarboxylates, such as lactate, pyruvate, and ketone bodies, across cell membranes [26]. It facilitates the movement of these molecules by coupling their transport with a proton gradient. This transport mechanism is important for energy metabolism and cellular homeostasis maintenance. The exact implications of SLC16A4 in various diseases and conditions require further investigation.

SLC16A3, also known as monocarboxylate transporter 4 (MCT4), is a protein belonging to the solute carrier family 16 (SLC16) of transporters. It is a transmembrane protein composed of 12 transmembrane domains and intracellular N- and C-termini. SLC16A3 is predominantly located on the plasma membrane of various tissues and cell types, including skeletal muscle, heart, brain, kidneys, and intestines [27]. SLC16A3 is known to play a significant role in tumor metabolism in the context of cancer. Cancer cells often exhibit altered metabolism, characterized by increased glycolysis even in the presence of sufficient oxygen, a phenomenon known as the Warburg effect. SLC16A3 is upregulated in several types of cancer and is responsible for the efflux of lactate from cancer cells [28, 29]. By promoting lactate release, SLC16A3 contributes to tumor microenvironment acidification and serves as an energy source for adjacent cancer cells. Increased *SLC16A3* expression in cancer cells causes accumulation of lactate in the tumor microenvironment, which results in an acidic pH [28]. This acidic environment affects various aspects of tumor progression, including immune evasion, angiogenesis, and metastasis [30–32]. Targeting SLC16A3 had gained attention as a potential therapeutic strategy in cancer treatment [33, 34]. By inhibiting or modulating the activity of SLC16A3, it may be possible to disrupt lactate export, alter the tumor microenvironment, and potentially enhance the effectiveness of other cancer treatments [26, 35, 36]. Further research is needed to fully understand the mechanisms by which SLC16A3 contributes to tumor metabolism and the complex interactions within the tumor microenvironment. However, targeting SLC16A3 demonstrated potential for improving cancer therapy outcomes [37].

SLC12A8, also known as electroneutral potassium chloride cotransporter 2, is a protein that belongs to the solute carrier family 12 (SLC12) of transporters. SLC12A8 is involved in the regulation of various physiological processes, although its precise functions remain under investigation. A previous study indicated that it plays a role in the maintenance of blood pressure and fluid balance in the body [38]. In addition to its involvement in ion transport, SLC12A8 is involved in certain cancers [39]. Altered *SLC12A8* expression levels have been observed in cancer cells, indicating its potential role in cancer biology [40]. However, the exact mechanisms by which SLC12A8 contributes to cancer development and progression remain unknown. Further studies are needed to elucidate the precise functions of SLC12A8 in the context of cancer and determine its significance as a potential therapeutic target.

SLC25A15, also known as mitochondrial ornithine transporter 1, has not been extensively studied in the context of cancer [41]. Most studies on SLC25A15 have focused on its role in the transport of ornithine and its association with the genetic disorder hyperornithinemia–hyperammonemia–homocitrullinuria syndrome [42]. The exact involvement of SLC25A15 in cancer remains unknown; however, alterations in mitochondrial transporters and metabolism have been implicated in various aspects of cancer biology, including cell survival, proliferation, and drug resistance. Mitochondria have been reported to play a crucial role in energy production and metabolism within cells, and dysregulation of mitochondrial function can profoundly affect cancer development and progression [43, 44]. While specific studies on SLC25A15 in cancer are limited, exploring its potential role in tumor biology is important. Further research is needed to investigate whether SLC25A15 alterations or dysregulation are associated with specific cancer types and elucidate the mechanisms by which SLC25A15 may impact tumor development and progression.

The prognostic risk scoring model demonstrated good performance in predicting patients’ survival and prognosis, with a predictive accuracy ranging from 0.675 to 0.85. Establishment of this prognostic risk scoring model provided a useful tool for clinicians to assist in decision-making. It helped them better evaluate patients’ prognoses and develop appropriate treatment plans. However, further research and validation are needed to ensure the applicability and stability of this model in different populations and disease types.

In addition, SLC25A29 gained our attention due to its significant correlation with OS and staging and its potential association with angiogenesis. SLC25A29, also known as the mitochondrial carnitine–acylcarnitine translocase, is a protein that plays a crucial role in the transport of acylcarnitines across the inner mitochondrial membrane [18]. Most studies on SLC25A29 have focused on its role in inherited metabolic disorders. However, specific research available on SLC25A29 is limited, and its involvement in cancer has not been extensively studied. In this study, the downregulation of *SLC25A29* in tumor tissue and its lower expression in endothelial cells in LUAD tissues compared with adjacent normal tissues were confirmed through various experiments, including RT-qPCR, western blotting, and immunohistochemistry. *SLC25A29* knockdown using siRNA promoted HUVEC migration and cell proliferation and reduced apoptosis, indicating its involvement in angiogenesis. Furthermore, this study investigated the potential regulatory factors affecting *SLC25A29* expression. ChIP-qPCR experiments have confirmed the critical regulatory role played by H3K14la and H3K18la modifications in the promoter region of *SLC25A29*.

So far, we could only find one study related to the role of *SLC25A29* in cancer in the Pubmed database [45]. This study may provide some preliminary information, but further research is necessary to comprehensively understand the function and potential impact of *SLC25A29* in cancer. These findings highlighted the potential importance of *SLC25A29* in cancer progression and indicated its potential role as a biomarker or therapeutic target.

However, this study has certain limitations. Retrieving datasets that are as comprehensive in detecting genes, have a sufficient number of samples, and include matched clinical information as the TCGA database is difficult with currently available databases. Therefore, this predictive model still requires more clinical data for validation. In addition, the specific regulatory mechanisms of *SLC25A29* need further exploration. Furthermore, deeper and clearer understanding of the molecular functions of the SLC family is needed.

## Conclusion

This study conducted data analysis and established a risk scoring model that consisted of five independent prognostic genes. The combination of risk scores and clinical–pathological variables in a nomogram provided an intuitive and accurate method for predicting the 1-year, 3-year, and 5-year survival rates of patients. Overall, this study highlighted the effects of SLC family genes on predicting patient OS. In addition, it is highlighted the importance of *SLC25A29* in angiogenesis and revealed a novel mechanism involving lactic acid-mediated histone modification in the regulation of *SLC25A29* expression.

### Electronic Supplementary Material

Below is the link to the electronic supplementary material.

Supplementary Material 1

## Data Availability

The datasets presented in this study can be found in online repositories. The names of the repository/repositories and accession number(s) can be found in the article/Supplementary Material.

## Abbreviations

GO: Geneontology
KEGG: Kyoto encyclopedia of genes and genomes
LASSO: Least absolute shrinkage selection operator
LUAD: Lung adenocarcinoma
MF: Molecular function
NSCLC: Non-small cell lung cancer
OS: Overall survival
PC: Principal components
PCA: Principal component analysis
ROC: Receiver operating characteristic curves
TCGA: The Cancer Genome Atlas
t-SNE: The t-distributed stochastic neighbor embedding
ECAR: Extracellular acidification rate
KLA: Lysine lactylation
CM: Conditioned medium
HUVEC: Human Umbilical Vein Endothelial Cells
DEG: Differential expressed genes
SLC: Solute carrier family
