# Supplementary material for: Comprehensive bioinformatics analysis of the solute carrier family and preliminary exploration of SLC25A29 in lung adenocarcinoma
Source: Cancer Cell Int. 2023 Sep 29;23:222. doi: 10.1186/s12935-023-03082-7 (PMC10543265; doi:10.1186/s12935-023-03082-7)
Supplement: Supplementary file 1 — Supplementary Material 1 [file 12935_2023_3082_MOESM1_ESM.docx]

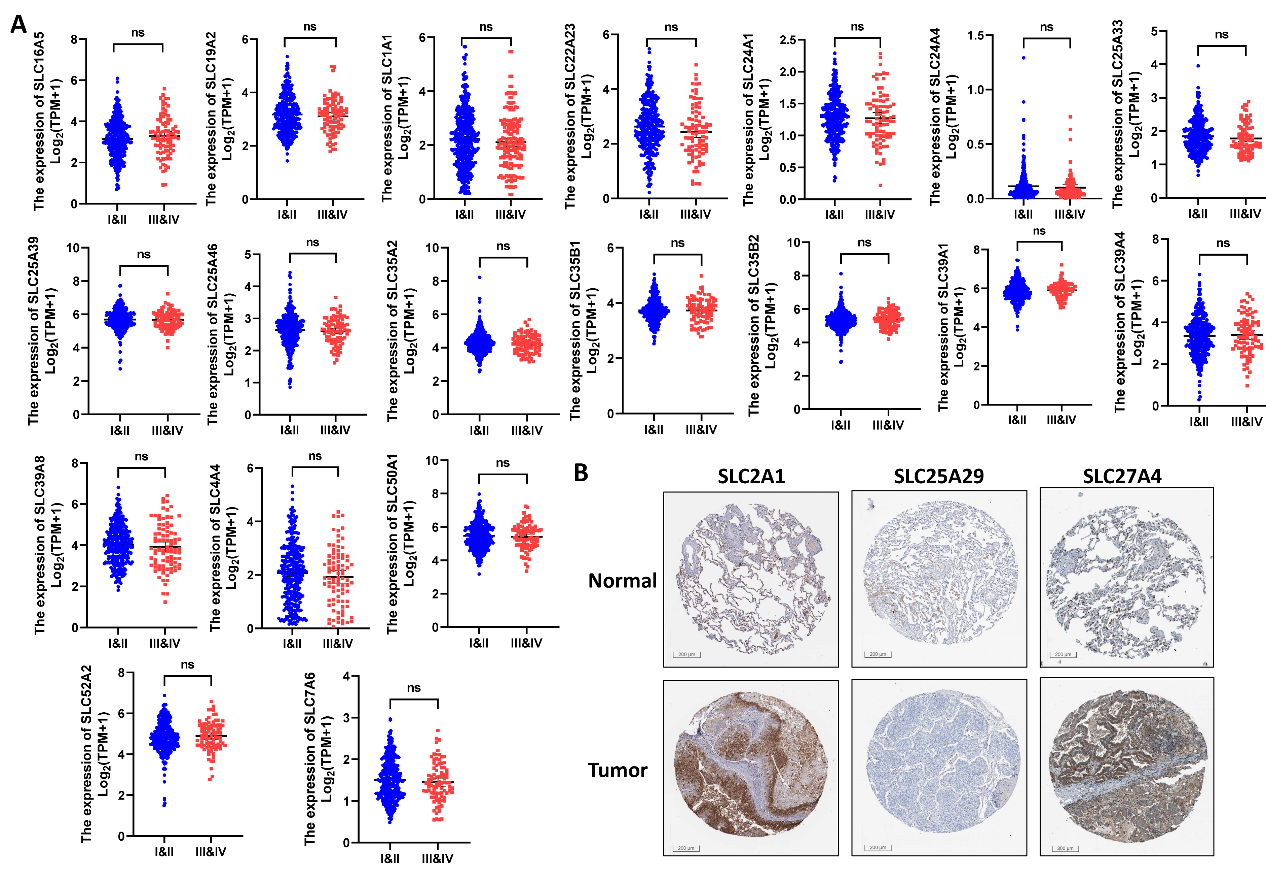


**Supplementary Figure 1.** Clinical relevance and expression of important genes of SLC members. (A) Correlation between survival related SLC gene and clinical stage. (B) Protein expression of SLC2A1, SLC25A29 and SLC27A4 in HPA database. ns, nonsignificant, *P < 0.05, **P < 0.01, ***P < 0.001, ****P < 0.0001.


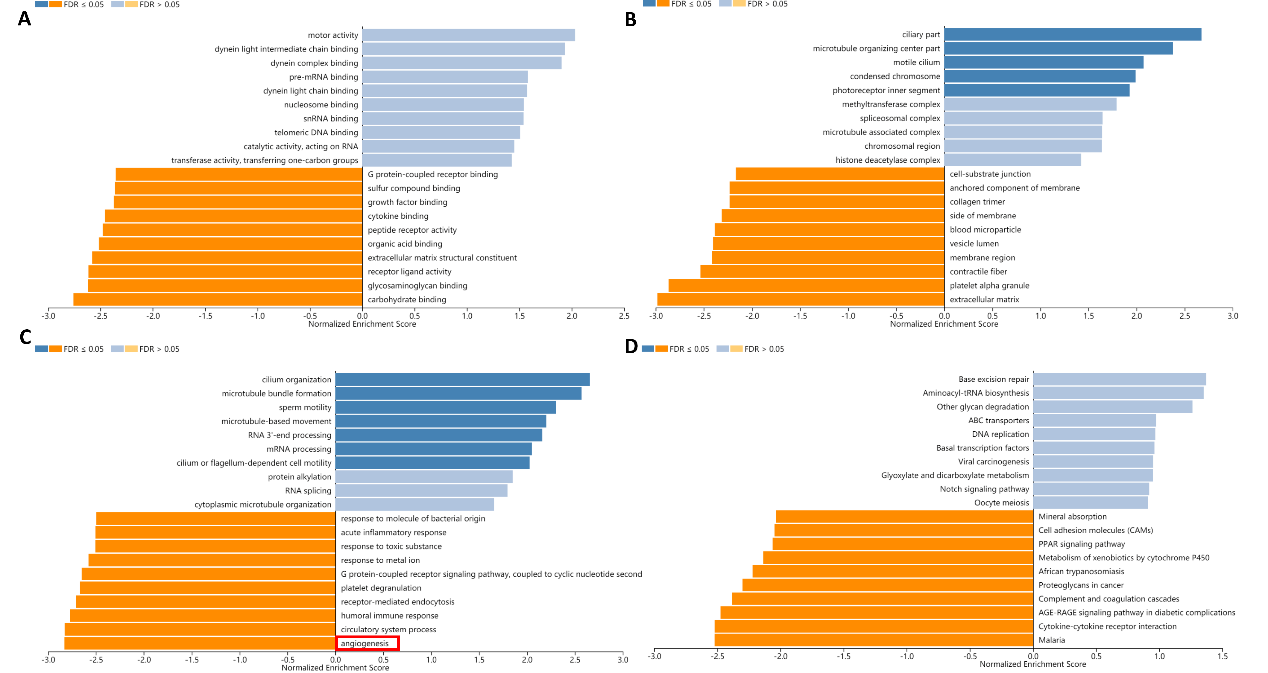


**Supplementary Figure 2.** GO and KEGG enrichment analysis of SDEGs. Molecular function (A), cell components (B), biological processes (C) and KEGG (D) enrichment analysis of SDEGs. Blue represented pathway activation, while orange represented pathway inhibition.


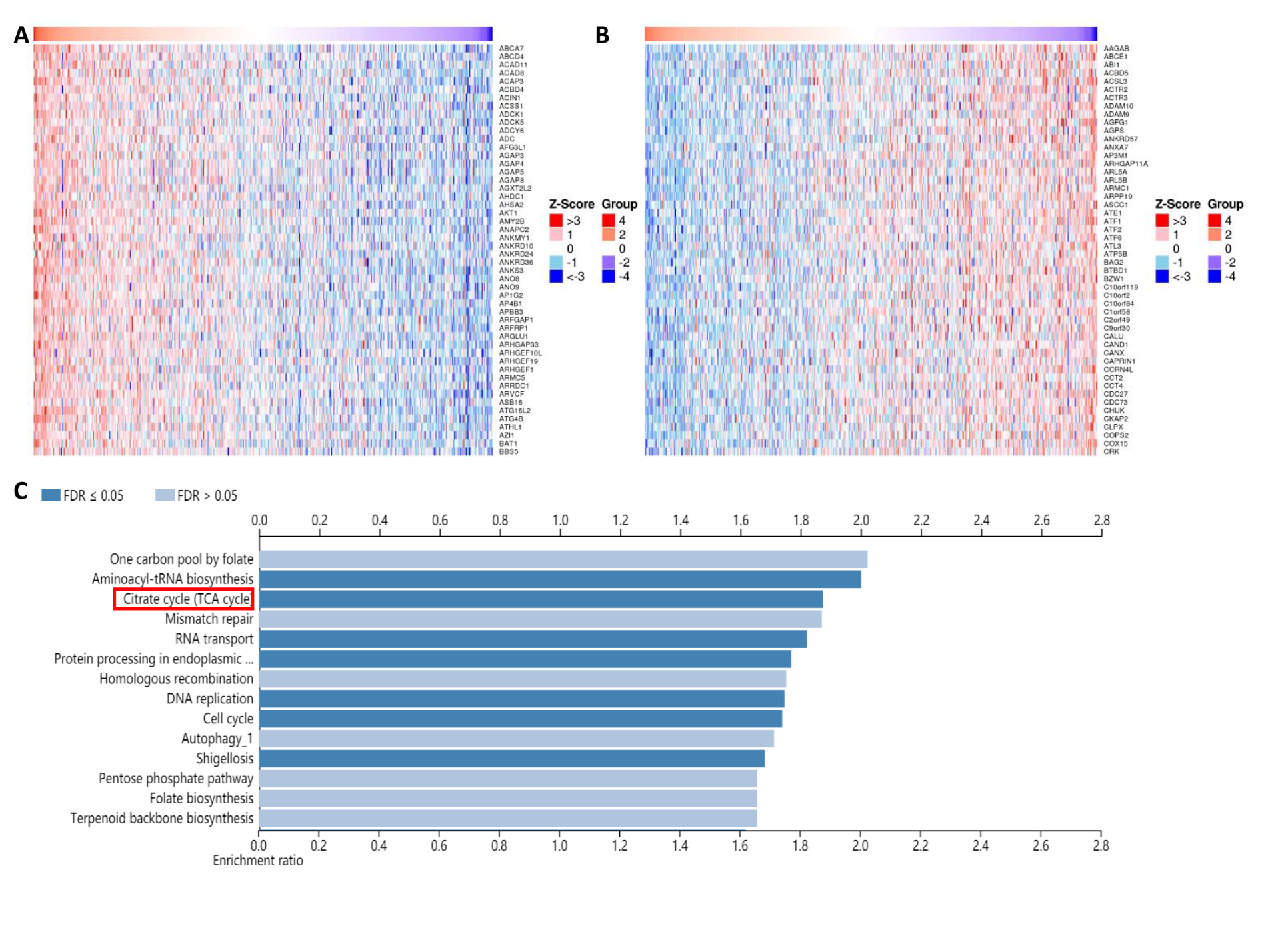


**Supplementary Figure 3.** Correlation analysis of SLC25A29 in TCGA database. (A and B) Heat maps of partial genes positively or negatively associated with SLC25A29. (C) Enrichment analysis of genes negatively associated with SLC25A29.


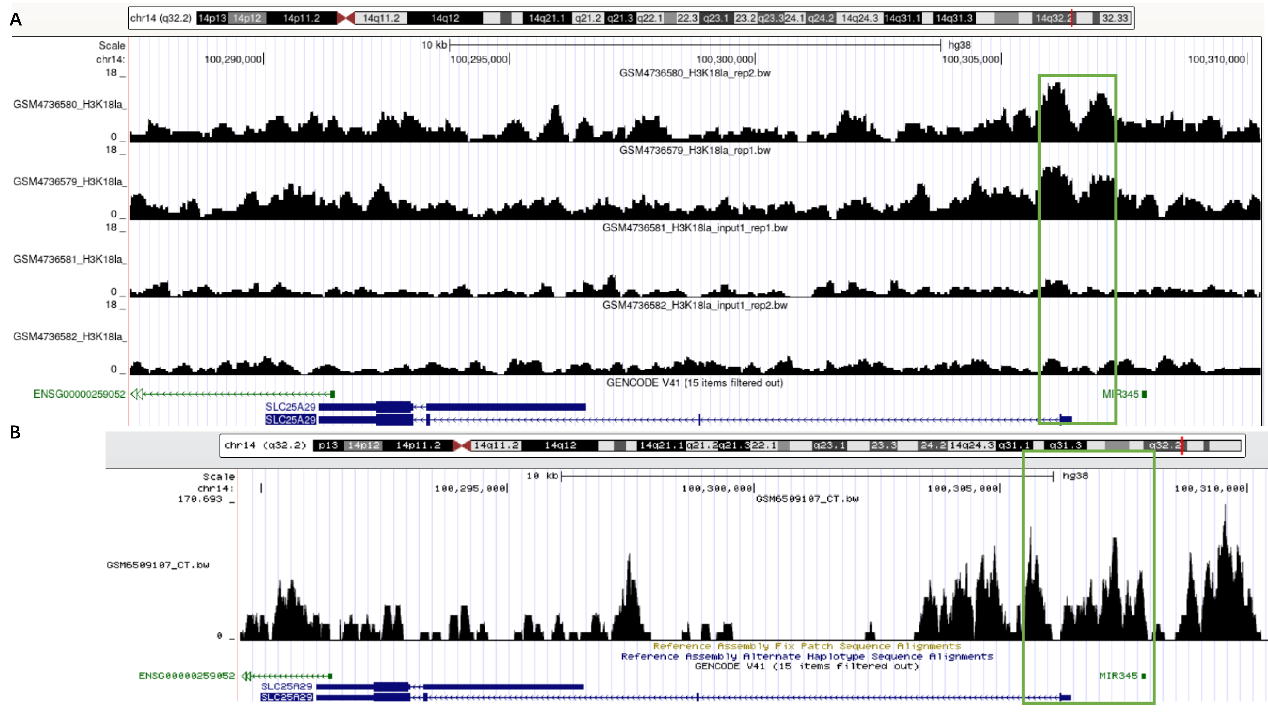


**Supplementary Figure 4.** Distribution statistics of peaks in SLC25A29 genome region in chip sequence. (A) Distribution statistics of H3K18la peaks in SLC25A29 genomic region in ocular melanoma. (B) Distribution statistics of H3K14la peaks in SLC25A29 genomic region in HUVEC.

**Supplementary table 1. Univariate analysis results of 13 genes in the TCGA dataset.**

| **Gene** | **HR** | **HR.95L** | **HR.95H** | **p value** |
| --- | --- | --- | --- | --- |
| **SLC16A3** | **1.0223** | **1.0129** | **1.0319** | **0.0000** |
| **SLC15A1** | **1.1155** | **1.0634** | **1.1702** | **0.0000** |
| **SLC25A15** | **1.0717** | **1.0392** | **1.1053** | **0.0000** |
| **SLC16A4** | **1.0168** | **1.0069** | **1.0267** | **0.0008** |
| **SLC16A6** | **1.0658** | **1.0201** | **1.1135** | **0.0043** |
| **SLC22A23** | **0.9450** | **0.9077** | **0.9838** | **0.0058** |
| **SLC12A8** | **1.0409** | **1.0104** | **1.0724** | **0.0083** |
| **SLC24A3** | **0.9270** | **0.8658** | **0.9925** | **0.0294** |
| **SLC13A5** | **1.1436** | **1.0064** | **1.2995** | **0.0397** |
| **SLC25A14** | **0.8344** | **0.7015** | **0.9925** | **0.0408** |
| **SLC16A7** | **1.0828** | **1.0031** | **1.1689** | **0.0415** |
| **SLC18A2** | **0.7198** | **0.5245** | **0.9878** | **0.0417** |
| **SLC16A13** | **1.0700** | **1.0002** | **1.1447** | **0.0495** |

**Supplementary table 2. Multivariate analysis results of 5 genes in the TCGA dataset.**

| **id** | **HR** | **HR.95L** | **HR.95H** | **P value** |
| --- | --- | --- | --- | --- |
| **SLC12A8** | **1.037790516** | **1.006484324** | **1.070070472** | **0.0176** |
| **SLC16A3** | **1.021116852** | **1.011034326** | **1.031299926** | **<0.001** |
| **SLC16A4** | **1.013797987** | **1.00322809** | **1.024479248** | **0.0104** |
| **SLC16A7** | **1.096435989** | **1.012482169** | **1.187351161** | **0.0235** |
| **SLC22A23** | **0.964570124** | **0.928021475** | **1.002558184** | **0.0672** |
| **SLC25A15** | **1.067559877** | **1.035311087** | **1.100813181** | **<0.001** |

**Supplementary table 3. Univariate analysis results of risk score and clinicopathological in the TCGA dataset.**

| **Gene** | **HR** | **HR.95L** | **HR.95H** | **P value** |
| --- | --- | --- | --- | --- |
| **Age** | **1.0109** | **0.9927** | **1.0294** | **0.2438** |
| **Gender** | **0.9844** | **0.6915** | **1.4013** | **0.9304** |
| **Stage** | **1.7192** | **1.4471** | **2.0424** | **<0.001** |
| **Risk score** | **1.2106** | **1.1459** | **1.2789** | **<0.001** |

**Supplementary table 4. Multivariate analysis results of risk score and clinicopathological in the TCGA dataset.**

| **Gene** | **HR** | **HR.95L** | **HR.95H** | **P value** |
| --- | --- | --- | --- | --- |
| **Age** | **1.0191** | **1.0008** | **1.0378** | **0.0409** |
| **Gender** | **1.0204** | **0.7148** | **1.4566** | **0.9114** |
| **Stage** | **1.7037** | **1.4207** | **2.0432** | **<0.001** |
| **Risk score** | **1.1668** | **1.1017** | **1.2358** | **<0.001** |

**Supplementary table 5. A brief list of metabolic substrates transported by survival related SLC.**

| Gene | Aliases | Type of Substrates |
| --- | --- | --- |
| SLC1A1 | Sodium-Dependent Glutamate/Aspartate Transporter 3 | Amino acids |
| SLC4A4 | Sodium Bicarbonate Cotransporter | Bicarbonate |
| SLC16A5 | Monocarboxylate Transporter 5 | Monocarboxylate |
| SLC25A29 | Mitochondrial Basic Amino Acids Transporter | Amino acids |
| SLC12A4 | Electroneutral Potassium-Chloride Cotransporter 1 | Inorganic ion |
| SLC24A3 | Sodium Calcium Exchanger | Inorganic ion |
| SLC22A23 | Chromosome 6 Open Reading Frame 85 | Inorganic ion |
| SLC39A8 | BCG-Induced Integral Membrane Protein In Monocyte Clone 103 | Inorganic ion |
| SLC24A1 | Retinal Rod Na-Ca+ K Exchanger | Inorganic ion |
| SLC19A2 | High Affinity Thiamine Transporter | Vitamin |
| SLC7A6 | Y (+) L-Type Amino Acid Transporter 2 | Amino acids |
| SLC24A4 | Na (+)/K (+)/Ca (2+)-Exchange Protein 4 | Inorganic ion |
| SLC25A33 | Pyrimidine Nucleotide Carrier 1 | Nucleotides |
| SLC27A4 | Fatty Acid Transport Protein 4 | Fatty Acid |
| SLC35A2 | UDP-Galactose Transporter | Carbohydrate |
| SLC25A46 | - | - |
| SLC25A39 | Probable Mitochondrial Glutathione Transporter | Small peptide |
| SLC35B1 | Endoplasmic Reticulum ATP/ADP Translocase | Carbohydrate |
| SLC39A1 | Zinc-Iron-Regulated Transporter-Like 1 | Inorganic ion |
| SLC35B2 | Adenosine 3'-Phospho 5'-Phosphosulfate Transporter | Inorganic ion |
| SLC52A2 | Riboflavin Transporter 3 | Nucleotides |
| SLC50A1 | Stromal Cell Protein | Carbohydrate |
| SLC2A1 | Glucose Transporter Type 1 | Carbohydrate |
| SLC39A4 | Zinc-Iron-Regulated Transporter-Like 4 | Inorganic ion |
